# Surface Engineering of Liposomes for Stealth Behavior

**DOI:** 10.3390/pharmaceutics5040542

**Published:** 2013-10-25

**Authors:** Okhil K. Nag, Vibhudutta Awasthi

**Affiliations:** Department of Pharmaceutical Sciences, College of Pharmacy, University of Oklahoma Health Sciences Center, 1110 North Stonewall Avenue, Oklahoma City, OK 73117, USA; E-Mail: okhil-nag@ouhsc.edu

**Keywords:** stealth liposomes, complement proteins, poly(ethylene glycol), lipopolymer

## Abstract

Liposomes are used as a delivery vehicle for drug molecules and imaging agents. The major impetus in their biomedical applications comes from the ability to prolong their circulation half-life after administration. Conventional liposomes are easily recognized by the mononuclear phagocyte system and are rapidly cleared from the blood stream. Modification of the liposomal surface with hydrophilic polymers delays the elimination process by endowing them with stealth properties. In recent times, the development of various materials for surface engineering of liposomes and other nanomaterials has made remarkable progress. Poly(ethylene glycol)-linked phospholipids (PEG-PLs) are the best representatives of such materials. Although PEG-PLs have served the formulation scientists amazingly well, closer scrutiny has uncovered a few shortcomings, especially pertaining to immunogenicity and pharmaceutical characteristics (drug loading, targeting, *etc.*) of PEG. On the other hand, researchers have also begun questioning the biological behavior of the phospholipid portion in PEG-PLs. Consequently, stealth lipopolymers consisting of non-phospholipids and PEG-alternatives are being developed. These novel lipopolymers offer the potential advantages of structural versatility, reduced complement activation, greater stability, flexible handling and storage procedures and low cost. In this article, we review the materials available as alternatives to PEG and PEG-lipopolymers for effective surface modification of liposomes.

## 1. Introduction

Over the past few decades, liposomes have acquired wide acceptance as the nanocarriers of choice for pharmaceutical applications [1,2]. Phospholipids are the main constituent of liposomes. The presence of a hydrophobic tail and a hydrophilic head group in phospholipids confers them with the ability to organize into spherical bilayer orientations in aqueous media. Thus, liposomes have a hydrophobic bulk membrane and a hydrophilic inner space. This enables them to entrap water-soluble, as well as lipophilic substances, while remaining dispersed in the aqueous environment. These attributes make liposomes a unique nano-vehicle for the delivery of biomedical agents.

Liposomes having long-circulating properties have especially drawn considerable attention for prolonged drug persistence in the body, blood pool imaging and targeted delivery of drugs. These applications primarily required liposomes to remain invisible to the normal clearance mechanisms, particularly by the organs of the mononuclear phagocyte system (MPS). This desired invisibility is constituted in the liposomes by decorating the outer surface of the liposomes with stealth-imparting polymeric substances [3,4]. Without being stealth, the conventional liposome surface is strongly affected by physical interactions with specific circulating proteins in blood in a phenomenon commonly referred to as opsonization. The opsonized liposomes could be seen as the particles tagged for uptake by MPS and subsequent clearance. These opsonizing proteins include complement proteins (C3, C5, *etc.*), laminin, fibronectin, C-reactive protein, type I collagen and immunoglobulins. What triggers the activation of complement proteins after liposome administration is not completely understood, but the process culminates into a cascade of inflammatory and complex adverse reactions that manifest as symptoms of transient cardiopulmonary distress [5,6,7]. Even though the process of opsonization is natural and is required for the innate immune response to clear pathogens, its inadvertent activation challenges the ability of the liposomes to stay in the blood pool. The efforts to develop polymer-based stealth technologies are directed towards overcoming this challenge. In essence, the liposomes are modified with biocompatible natural or synthetic materials to reduce the interaction of opsonizing proteins with the liposome surface.

Since the early 1990s, the development of various active materials for the surface engineering of liposomes and other nanomaterials has progressed much faster than before, mainly because of an urgent need for developing long-circulating vehicles for drugs in medicine. With a renewed interest in drug delivery using nano-technologies, this review will encompass the materials and technologies developed to accomplish effective surface modification of the liposomes.

## 2. Natural *versus* Synthetic Materials for Surface Modification

The use of modified and unmodified dextrans [8], pullulan [9] and gangliosides [10] has been considered to impart stealth properties to the liposomes. Of these, gangliosides garnered special attention. In fact, the use of gangliosides for attaining stealth behavior in liposomes precedes the advent of synthetic polymers for this purpose. Gangliosides are sialic acid-containing glycosphingolipids that are present exclusively in the outer leaflet of the plasma membrane. The role played by surface gangliosides in prolonged circulation of native red blood cells led to the application of their derivatives for the modification of the liposome surface. As such, the inclusion of monosialoganglioside (G_M1_) increased the circulation half-life of liposomes [11] and enhanced their accumulation in tumor volume, while reducing the uptake in liver and spleen [12]. The level of MPS uptake of G_M1_-modified liposomes correlates with the concentration of G_M1_ in the liposomes bilayer, and the sialic acid moiety plays an important role in preventing MPS uptake [13]. G_M1_ is characterized by a bulky hydrophilic group that not only provides a steric barrier, but also shields its anionic charge, which reduces the charge-based interactions with opsonizing proteins [12,14]. An alternative explanation is based on its binding to dysopsonins, which reduces the recognition of liposomes by MPS [15]. Despite these early successes, the use of gangliosides for prolonging the circulation persistence of liposomes fell in favor of the relatively inexpensive and more flexible synthetic poly(ethylene glycol) (PEG). For further advantages and disadvantages of the natural materials, the readers may refer to a previous review [16].

**Figure 1:**
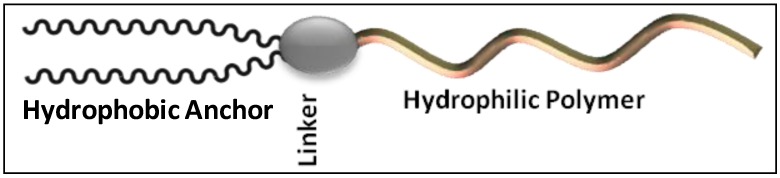
The stealth lipopolymers are characterized by a hydrophobic anchor (phospholipid, fatty acid or cholesterol), a linker (phosphate, ester, amide, *etc.*) and a hydrophilic polymer (poly(ethylene glycol), polyamino acid, *etc.*).

As a logical replacement of natural materials, various synthetic polymers have emerged that have been exploited to achieve the prolonged circulation time of the liposomes. The key common characteristics of the most successful polymers include biocompatibility, ease of synthesis, low fouling, flexibility and aqueous/organic solubility. For use in the constitution of liposomes, these polymers are converted into lipopolymers by attaching a lipophilic portion to their inherently hydrophilic domain (Figure 1). The resultant lipopolymers differ in their ionic charge and hydrophilic/lipophilic balance. In the following text, we discuss the lipopolymeric substances that have been historically used and some newly developed substances for the surface modification of liposomes.

## 3. Poly(Ethylene Glycol)-Linked-Lipids (PEG-Lipids)

PEG-based lipopolymers have brought the most important breakthrough in the development of stealth technologies. This is mainly because PEG is non-ionic, low fouling and possesses high solubility in both aqueous and organic media. The solubility of PEG in organic media not only enables the synthesis of PEGylated lipids, but also facilitates the formulation of stealth liposomes, which invariably requires solubilization of PEG-lipopolymer and other lipids in organic solvents. PEG is also characterized by excellent biocompatibility, lack of toxicity, low immunogenicity and antigenicity and good excretion kinetics [17,18,19,20,21]. In addition, PEG can be synthesized with a wide range of molecular weight (MW, 400 Da to 50 kDa) with a low polydispersity index (PDI, below 1.1) and different end-group functionalities [22,23,24]. The resultant PEGylated liposomes are arguably the best engineered long-circulating nanocarriers, demonstrating circulation half-lives of 15–24 h in rodents and as high as 45 h in humans [25]. As such, several PEGylated liposome products have reached clinical usage [26]. PEG inhibits non-specific and specific protein interaction on the liposomes surface, and their mechanism of action has been reviewed in several instances [15,25,27,28]. It is believed that PEG sterically keeps the approaching plasma proteins away from the liposomes surface by coating it with PEG in mushroom or brush conformations. The actual conformation of PEG depends on the grafting density and MW of PEG [15,29,30]. A brush-like PEG conformation dominates at high PEG grafting, whereas a mushroom conformation is mostly observed at low grafting density [31].

**Figure 2:**
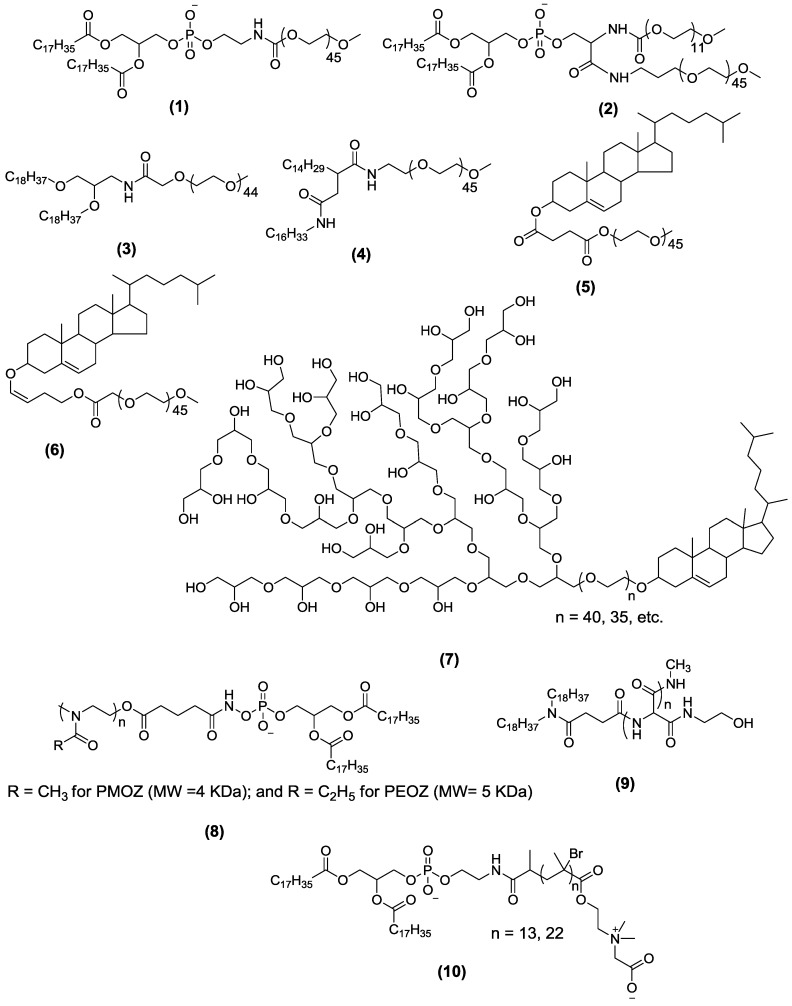
Chemical structures of representative lipopolymers containing different polymers, linkers and acyl chains. For more details about the degree of polymerization or values of *n*, the reader may refer to the references mentioned in the text. DSPE-PEG_2000_ (distearoyl-poly(ethylene glycol)) (**1**); PEG_2000_-DSPE-PEG_500_ (**2**); 1,2-distearyloxypropyl-3-amine-PEG_2000_ (**3**); hexadecylcarbamoylmethyl hexadecanoate-PEG_2000_ (**4**); cholesteryl hemisuccinate-PEG_2000_ (**5**); photocleavable cholesteryl-PEG_2000_ (**6**); cholesterol-hyperbranched polyglycerol (**7**); poly(2-methyl-2-oxazoline) (PMOZ)- or poly(2-ethyl-2-oxazoline) (PEOZ)-DSPE (**8**); poly(hydroxyethyl-l-asparagine)-succinyldioctadecylamine (**9**); DSPE-poly(2-*tert*-butoxy-*N*-(2-(methacryloyloxy)ethyl)-*N*,*N*-dimethyl-2-oxoethanamonium) (**10**).

The PEG-based lipopolymers are characterized by a PEG chain of variable length or architecture attached to the acyl (lipids) moiety through a linker, such as phosphate ester, carboxylate ester, amide, disulfide or ether linkage [32,33,34,35,36,37]. The acyl groups act as anchors embedded in the liposome bilayer, while the PEG chains localize towards the aqueous environment. The acyl chain length and architecture have a direct influence on the physical state of the lipid assemblies—lamellar *versus* micellar [38]. It also controls the rate and extent of inclusion of PEG lipopolymer in the liposome membrane [39,40]. A comparison of the effects of the ceramide (CER) acyl chain length (C8 through C24) on the pharmacokinetics of vincristine-loaded liposomes (sphingomyelin/cholesterol/PEG-CER) demonstrated that longer acyl chains on the PEG_2000_-CER were associated with longer circulation lifetimes and, consequently, higher plasma vincristine concentrations [41]. The short-chain ceramides rapidly partitioned from the vesicles after intravenous administration, whereas the PEG-ceramides with longer acyl chains partitioned slowly, because of the stronger anchoring properties in the liposome bilayer. However, the increase in the acyl chain length results in a reduction of the magnitude of liposome PEGylation; PEG_2000_-dimyristoylglyceride incorporated in the liposomes to the extent of 80 mol% *versus* 57 mol% of PEG_2000_-distearoylglyceride [40]. The choice of linker groups may also impact the overall behavior of the modified liposomes. For example, ester, vinyl ether and acetal linkages are highly susceptible to hydrolysis in biological media, whereas the presence of a negatively-charged phosphate linker may activate the complement [42,43]. Based on the PEG architecture, linker and acyl moieties (Figure 2), we categorize the PEG-lipids into the following groups.

### 3.1. PEG-Phospholipids

The class of PEG-phospholipids is populated by linear chain methoxy-PEG covalently bonded to the polar head group of phospholipids, such as distearoyl- (**1**), dipalmitoyl-, and dimyristoyl-phosphatidylethanolamine (DSPE, DPPE and DMPE, respectively). The influence of these PEG-phospholipids on circulation persistence is dependent on both the PEG chain-length and their grafting density relative to the total lipid constitution [44,45]. In general, long PEG chains provide a better steric barrier than short PEG chains. For example, the blood levels of liposomes increased from 16% to 28% when the MW of PEG was increased from 750 to 5000 Da [45]. PEG modification forms a fixed aqueous layer thickness (FALT) around the surface of the liposome [46]. FALT increases with the increase in PEG MW, which positively correlates with the duration of the persistence of liposomes in circulation [47]. In addition, liposomes modified with mixed PEGs having long, as well as short chains (PEG_2000_ and PEG_500_) demonstrate thicker FALT compared to that observed in the liposomes modified with a single PEG chain [48,49,50]. Interestingly, 1,2-distearoyl-*sn*-glycero-3-phosphoethanolamine-PEG^2^ (**2**), having two different PEGs arms of MW 2000 and 500 Da in the same molecule, maintains a longer circulation time, with a larger FALT value and a higher contact ability with tumor cells compared to the liposomes PEGylated with regular PEG-lipids [47]. The steric stabilization afforded by PEG-phospholipids is also influenced by the size of the liposomes. There exists a maximum liposome size (~275 nm) beyond which the stealth property of PEG-liposomes is significantly compromised, and its distribution is characterized by high MPS accumulation [51]. In addition to the steric stabilization, PEG-phospholipids also influence the liposome permeability and the rate and mechanism of drug release [52]. For instance, the increase in DMPE-PEG level in the liposomes was found to significantly reduce the release rate constant of encapsulated guanosine [53]. The investigators also found that the mechanism of guanosine release changed from diffusion-controlled to interfacial-controlled, as the amount of PEG-phospholipid in the bilayer was increased, and the magnitude of the effect was directly dependent on the MW of PEG [53].

### 3.2. PEG-Non-Phospholipids

Although synthetic PEGs conjugated to phospholipids have successfully reached clinical applications, it is worth pointing out certain limitations of a phospholipid anchor. In order to anchor PEG at the surface of the liposomes, its conjugation to a phospholipid is not necessary. A new class of materials emerged in which PEG is linked to a non-phospholipid anchor. The goal has been to eliminate potential problems that have been attributed to phospholipid anchors in these amphiphiles [54]. The phosphoryl moiety of phospholipids carries a negative charge, which has been linked to hypersensitivity reactions and anaphylatoxin production observed with the usage of liposome products [43,55,56]. The net anionic charge of the liposomes has also been linked to the activation of platelets [57]. Such biological reactions are frequently seen, even with highly purified phospholipids, and are believed to be associated with complement activation [58,59,60,61]. Secondly, phospholipids are relatively unstable, being especially susceptible to enzymatic degradation [62]. Lastly, they are expensive, because of the costs associated with extraction, synthesis and storage of phospholipids [63,64]. Consequently, interest has grown recently in developing non-phospholipids and PEG-non-phospholipids amphiphiles, which have the potential to offer the significant advantages of structural versatility, reduced reactivity to complement proteins, greater stability, flexible handling and storage procedures and low cost [34,35,63,65,66,67]. Several amphiphiles, including those containing sphingolipids, fatty acids and their salts and derivatives, and their conjugates with derivatives of polymers, such as polyoxyethylene and polyglycerol, have been reported [68].

Heyes *et al.* designed a few novel PEG-lipids with the aim of preparing PEG-stabilized liposomal vesicles encapsulating plasmid DNA [34]. PEG_2000_ was linked to a 1,2-distearyloxypropyl-3-amine anchor using succinimide, amide and carbamate linkers (**3**). It was found that these PEG-lipids were more stable in liposomal formulation, less toxic upon systemic administration and could replace the previously used PEG-succinoyl diacylglycerols (PEG-S-DSGs) for the same purpose [34]. We recently reported a novel PEG-conjugated hexadecylcarbamoylmethyl hexadecanoate (HDAS-PEG_2000_, **4**) as a PEG-phospholipid substitute for enhancing the circulation persistence of liposomes [35]. HDAS-PEG significantly reduced the liposome-induced complement activation (C4d, Bb and SC5b) and was found to be more effective than commonly used DSPE-PEG in this capacity. It was also relatively resistant to spontaneous desorption from the liposome surface upon dilution of the liposomes [35].

Cholesteryl-PEG (Chol-PEG) is another variant of PEG-non-phospholipid. The presence of large amounts of cholesterol in most liposome formulations makes cholesterol a natural choice as an anchor for PEG. Cholesterol enhances hydration of the lipid head group, stabilizes the membrane and improves the retention of hydrophilic drugs [69,70]. In the Chol-PEG conjugate, the PEG chain is attached to the 3-OH group of cholesterol via an ester or ether linkage. Because of cholesterol’s lipophilicity and structural compatibility with liposomal phospholipids, Chol-PEG is easily incorporated into the liposome membranes [71]. Studies have also demonstrated that Chol-PEG, like cholesterol, could work as a framework by reducing membrane fluidity, increasing the stability of the bilayer and controlling the drug permeability of the liposomal bilayer [72,73,74]. However, the effect of Chol-PEG_2000_ on drug-loading efficiency and biological half-life is determined by the total cholesterol content in the liposome formulation. At low total cholesterol content (hydrogenated soybean phosphatidylcholine (HSPC)/Chol 4:1 M ratio), both the loading efficiency of vinorelbine and the circulation half-life of modified liposomes decreased with increasing (from 3 mol% to 8 mol%) Chol-PEG_2000_ concentration, whereas the opposite effects were observed when Chol-PEG was increased in liposomes containing high total cholesterol (HSPC/Chol 3:1 M ratio) [75]. Cisplatin-loaded liposomes modified with Chol-PEG have been shown to exhibit prolonged circulation; after 12 h of administration, Chol-PEG_550/2000_-modified liposomes were found to maintain the cisplatin level in blood more than four times as much as compared to the unmodified liposomes [72].

Chol-PEG has also been used in creating liposomes, the contents of which could be released by cleaving the PEG chain (dePEGylation). For instance, PEG-cholesteryl methyl carbamate (PEG-CHMC) and PEG-cholesteryl hemisuccinate (PEG-CHEMS, **5**) are susceptible to esterase-mediated cleavage; the liposomes carrying these two lipopolymers are destabilized in the presence of esterase, enabling the release of encapsulated contents [76]. Similarly, liposomes containing ethers linked Chol-PEG,1'-(4'-cholesteryloxy-3'-butenyl)-ω-PEG_5000_ are susceptible to acid-induced content release [77]. Yet another modification is the liposomes containing photo-cleavable Chol-PEG (**6**) [67], which potentially appears to be more controllable than the acid/esterase-mediated cleavage. Because of the gradual cleavage of the PEG chain, liposomes modified with cleavable PEG-CHMC and PEG-CHEMS exhibit shorter prolongation of circulation persistence, but they have also been found to display a reduced “accelerated blood clearance” (ABC) phenomenon, which occurs upon repeated administration of PEG-lipids [78].

Despite being an attractive target for PEG-anchoring, it is noteworthy that liposomes modified with Chol-PEG exhibit shorter prolongation of circulation persistence than that observed with PEG-phospholipids. It has been speculated that this difference is probably due to the reduced conformational flexibility of PEG, since the cholesterol anchor in Chol-PEG could be located deeper (as compared to the phospholipid in the PEG-phospholipids) in the liposome membrane [79]. The deeper presence of the PEG chain has also been suggested as a cause of Chol-PEG’s tendency to perturb the lipid bilayer, resulting in relatively rapid release of entrapped drugs, especially at a high Chol-PEG density [79]. Therefore, a linker (L) has been introduced between Chol and PEG molecules (Chol-L-PEG) in some designs. For instance, a 1,4-diaminobutane linker in Chol-L-PEG_3400_ reduced the serum-induced release of encapsulated carboxyfluorescein and decreased the adsorption of serum proteins comparable to that observed with 5 mol% of PEG-DSPE-PEG, but better than that observed with Chol-PEG_3400_ [80].

### 3.3. Hyperbranched Polyglycerol (hbPG)-Lipids

Hyperbranched polyglycerol (hbPG) offer several advantages over PEG. They are thermally and oxidatively more stable than linear PEG, while maintaining the same level of resistance against protein adsorption [81]. In addition, hbPG can be synthesized from various epoxides via anionic ring-opening polymerization in a controlled manner using cholesterol directly as an initiator. The use of cholesterol as an initiator eliminates laborious coupling chemistry needed to link phospholipids or cholesterol anchors [82]. Recently, Hofmann *et al.* reported the synthesis of a series of hyperbranched polyglycerol-based lipids, with 1,2-bis-*n*-tetradecyl glyceryl ether or cholesterol (**7**) as anchors. These lipids were introduced into the liposomes without compromising its structural integrity [83]. Apart from this early report, the application of hbPG in liposome surface engineering has not been well studied, and to the best of the authors’ knowledge, the stealth behavior of such hbPG-modified liposomes has not been studied *in vivo*. Nonetheless, some attractive properties of hbPG are suggestive of their promise. Their effects on complement activation, red blood cell aggregation, toxicology and whole blood viscosity study with hbPG are as good or better as compared to the other polymers with linear PEG [84]. Moreover, the multiple hydroxyl groups on hbPG offer the ability to attach various functionalities via “click” or conventional chemistry [82,83]. For example, hydrophobic (–CH(OH)C_16_H_31_), as well as PEG_350_ derivatization of free hydroxyls in the hbPG resulted in a polymer with a plasma expanding property similar to that of human serum albumin [85]. This material was not found to activate platelets or complement systems and was reported to have only a small effect on plasma viscosity. In mice, it exhibited a circulation half-life of 34 h, which could be controlled by manipulating the MW and the degree of PEG derivatization [85].

## 4. Drawbacks of PEG Lipopolymers as Stealth Materials

Despite the overall safe and effective performance of PEG-liposomes, there exist a few physicochemical and physiological consequences of liposome PEGylation. These issues create an opportunity for the development of other lipopolymers. Structurally, a PEG chain has hydrophilic, as well as hydrophobic characteristics. The hydrophobic character of PEG prevents the hydration of membrane phospholipid headgroups, resulting in the destabilization of liposomes and the possibility of drug leakage and poor loading [86,87]. To minimize PEG chain–chain interaction, the need for excess cholesterol in PEGylated liposomes has been suggested [88]. Without the presence of sufficient cholesterol, PEG-phospholipids tend to phase separate and aggregate [38,87,89,90]. PEGylation also has an impact on the stability of liposome preparation. It can restrain, but cannot exclude, the fusion of liposomes upon prolonged storage in aqueous conditions [91]. In general, long-term stability could be achieved by freezing or lyophilization (freeze-drying), but in the absence of any cryoprotectant, the reconstituted PEGylated liposomes exhibit a significant increase in their size and polydispersity [92].

PEG-liposomes have been found to induce immunogenic responses. The adverse reactions of PEG often occur through complement activation, which leads to hypersensitivity reactions [93,94]. These reactions are ascribed to the presence of negatively-charged phosphoryl groups in clinically used PEG-phospholipids [43,55,56], variability in size, the nature of acyl anchor, *etc.* [95]. PEG has also been found to induce anti-PEG antibodies of the IgM class. Because of the increasing use of PEG in pharmaceuticals, cosmetics and foods products, anti-PEG antibodies have been detected even in healthy individuals without any prior exposure to PEG [96,97]. Anti-PEG antibodies play a major role in the clearance of an intravenously-administered PEGylated liposome product, especially when repeatedly administered (the ABC phenomenon). The ABC phenomenon not only affects bioavailability, but also passive targeting and its resultant efficacy of the liposome-encapsulated drug [98]. Recently, this specific IgM antibody response has been found to recognize the interface between hydrophilic PEG and hydrophobic lipid [99], which again points to the importance of choosing the right linker between the two moieties.

Besides immunologic reactions, long-term toxicity studies of PEGylated preparations are limited in number, and the majority of them seem to ignore the biological fate of the PEG after the disintegration of PEGylated liposomes [100,101,102]. It is worth noting that PEGs with MW less than 400 Da are prone to alcohol dehydrogenase-catalyzed oxidation, resulting in toxic diacid and hydroxy acid metabolites [103]. Although the tendency of undergoing oxidation decreases with increasing MW, PEGs with a size exceeding the cut-off of renal clearance (30–50 kDa) are not desirable, because of their relative non-biodegradability [104,105].

## 5. PEG Substitutes in Lipopolymers for Surface Engineering of Liposomes

In spite of the gradual emergence of issues associated with PEG and PEG-phospholipids, the advantages of PEG as a stealth polymer cannot be dismissed. For more information about the merits and demerits of PEG, the reader may refer to a nice review by Knop *et al.* [102]. PEG has served as the gold standard in formulations requiring prolonged circulation. However, the search for potential alternatives to PEG is getting intense, some of which is related to commercial and intellectual property interests associated with newer products. A brief discussion of these alternatives is presented in this section.

### 5.1. Vinyl-Based Lipopolymers

Polyvinyl pyrrolidone (PVP) is a water soluble, chemically stable, non-toxic and biocompatible polymer [106,107,108]. Compared to PEG, PVP shows better stability under UV or ultrasound irradiation and can be synthesized by free-radical or controlled radical polymerization [106,109]. A coating of polymerized PVP on a silicone surface reduced the complement activation by 90% of that demonstrated by uncoated silicone [110]. Liposomes modified with palmitate-linked PVP were found to have increased circulation time in blood and decreased accumulation in liver; the protective efficacy of the polymer increased with an increase in the length of the acyl moiety and decreased with an increase in the MW of the polymer [111]. In contrast, the circulation persistence of liposomes coated with palmitate-modified polyvinyl alcohol (PVA) increased with the increasing MW of PVA [112]. The PVA coating also suppressed the aggregation and fusion of the serum-challenged liposomes. Overall, the prolonged circulation time of liposomes coated with 1.3 mol% PVA_20 kDa_ was found to be comparable to that of stealth liposomes prepared with 8 mol% of DSPE-PEG_2 kDa_ [112]. The same investigators also evaluated the accumulation of doxorubicin-loaded and PVA-coated liposomes in rat tumors. The passive targeting efficiency of the PVA-coated liposomes was found to be approximately five times greater than that of the uncoated liposomes [112]. Despite these early promising reports, vinyl-based polymers have not caught the attention of liposome researchers yet. It has also been found that because of their non-biodegradable nature, PVP with a MW > 25 kDa accumulate in the body [113].

### 5.2. Poly(2-Oxazoline)-Based Lipopolymers

Water-soluble polyoxazolines have been studied for various biomedical applications, because of their amphiphilic and self-assembling nature [114,115,116,117]. These polymers, with various chemical functionalities and architectures, can be synthesized by cationic ring-opening polymerization in a controlled manner [116,118,119,120]. Poly(2-methyl-2-oxazoline) (PMOZ)- and poly(2-ethyl-2-oxazoline) (PEOZ)-linked DSPE (**8**) have been investigated for surface modification of liposomes as an alternative to PEG-DSPE [121]. Liposomes containing 5 mol% of PMOZ/PEOZ-DSPE showed a circulation behavior similar to that of liposomes containing corresponding PEG-DSPE. The circulation half-life of these liposomes was >15 h. In addition, the liposomes also showed reduced accumulation in liver and spleen [121]. Similar results were reported in another study with PMOZ/PEOZ-DSPE [122]. A recent study reported the synthesis of folate-poly(2-ethyl-2-oxazoline)-distearoyl phosphatidylethanolamine (F-PEOZ-DSPE) for targeting the folate-receptor (FR) overexpressed on cancer cells [123]. These FR-targeted liposomes were loaded with doxorubicin and were shown to exhibit selective targeting, as well as polymer-mediated protection [123]. Although poly(2-oxazoline) with various chemical functionalities has been tested for drug delivery and cell membrane construction, basic biological and stability studies have been very few [124]. To establish it as a PEG alternative, poly(2-oxazoline)-modified liposomes need to be further studied for opsonization, complement activation and stability.

### 5.3. Poly (Amino Acid)-Based Lipopolymers

Synthetic poly- (amino acid) or PAA-based stealth liposomes are attractive because of their complete biodegradable nature, which reduces the risk of polymer accumulation in various organs, as has been described to occur in the case of the non-degradable polymers [25,125,126]. PAAs, such as polyglutamic acid (PGA), poly(hydroxyethyl-l-asparagine) (PHEA) and poly(hydroxyethyl-l-glutamine) (PHEG), have been used in drug delivery applications [127,128,129,130,131,132]. Liposomes modified with PHEG-succinyldioctadecylamine (PHEG-DODASuc) or PHEA-DODASuc (**9**) have been shown to possess an extended blood circulation half-life and low liver or spleen uptake [130,133]. It was shown that approximately 22% of the injected liposomes (coated with 7.5 mol% PHEG_4000_, PHEA_3000_ or PHEA_5000_) remained in circulation after 24 h of injection. Although the clearance rate of PAA-liposomes was found to be similar to that of PEG-liposomes, the former were found to exhibit a lesser ABC phenomenon [134]. In addition, PAAs in free, as well as liposome-associated form are degraded by proteases, which is beneficial for their complete elimination from the body [133]. The enzymatic degradation of PAA-coated liposomes could also be a potentially useful mechanism for triggering the release of the liposome contents [135]. However, it must be noted that PHEG-DODASuc and PHEA-DODASuc lipopolymers showed increased complement activation [129], which may limit their eventual utility as polymers for the surface engineering of liposomes.

### 5.4. Zwitterionic Lipopolymers

Liposomal surface hydration is considered to play a key role in protein adsorption through nonspecific interactions. Studies have shown that strong surface hydration increases the thickness of the fixed aqueous layer around the surface, which prevents interaction with protein [47,136]. All the polymers discussed so far bestow stealth property to liposomes mainly by enabling hydration through H-bonding. However, H-bonding is a relatively weak interaction. On the other hand, materials containing zwitterionic phosphobetaine, sulfobetaine or carboxybetaine moieties can bind water molecules via strong electrostatic interaction [137,138,139]. The polymers or surfaces having zwitterionic functionalities are known to be ultralow fouling materials, since they have very little tendency to adsorb proteins [140,141,142]. Recently, Cao *et al.* reported the synthesis of DSPE-conjugated poly(2-*tert*-Butoxy-*N*-(2-(methacryloyloxy)ethyl)-*N*,*N*-dimethyl-2-oxoethanamonium) (DSPE-PCB, **10**) to stabilize liposomes without cholesterol, which is commonly required in PEGylated liposomes [143]. The liposomes containing 5 mol% DSPE-PCB_5000_ exhibited good stability and resistance against aggregation. The circulation half-life of these liposomes was found to be better than, or as long as, that observed with liposomes containing corresponding PEG-phospholipids. The enhanced hydration and fluidity of the liposome membrane provided by the poly(zwitterionic) component accounted for prolonged circulation. In addition, the zwitterionic liposomes loaded with doxorubicin showed better therapeutic efficiency than that of Doxil^®^ [143]. Our own experience with zwitterionic *N*^1^-(2-aminoethyl)-*N*^4^-hexadecyl-2-tetradecylsuccinamide-linked poly [*N*-(carboxymethyl)-2-(isobutyryloxy)-*N*,*N*-dimethylethanamonium] lipopolymer (HDAS-SHP) has been very encouraging. We found that the surface superhydrophilicity imparted by HDAS-SHP completely masked the negative zeta potential of the liposomes, reduced complement activation and enhanced the circulation persistence of the liposomes [144]. However, due to the lack of common solvents to dissolve both the hydrophobic and hydrophilic blocks together, the synthesis of such zwitterionic lipopolymers poses a significant challenge.

## 6. Stealth Liposomes Manufacturing Techniques

There are three ways to modify a liposome surface with lipopolymers: (1) incorporating an amphiphilic conjugate of the polymer during liposome formation (pre-insertion); (2) inserting the polymer conjugate onto the surface of pre-formed liposomes (post-insertion); and (3) post-modification by chemically reacting a polymer to the exposed functionalities on the liposome surface.

### 6.1. Pre-Insertion

**Figure 3:**
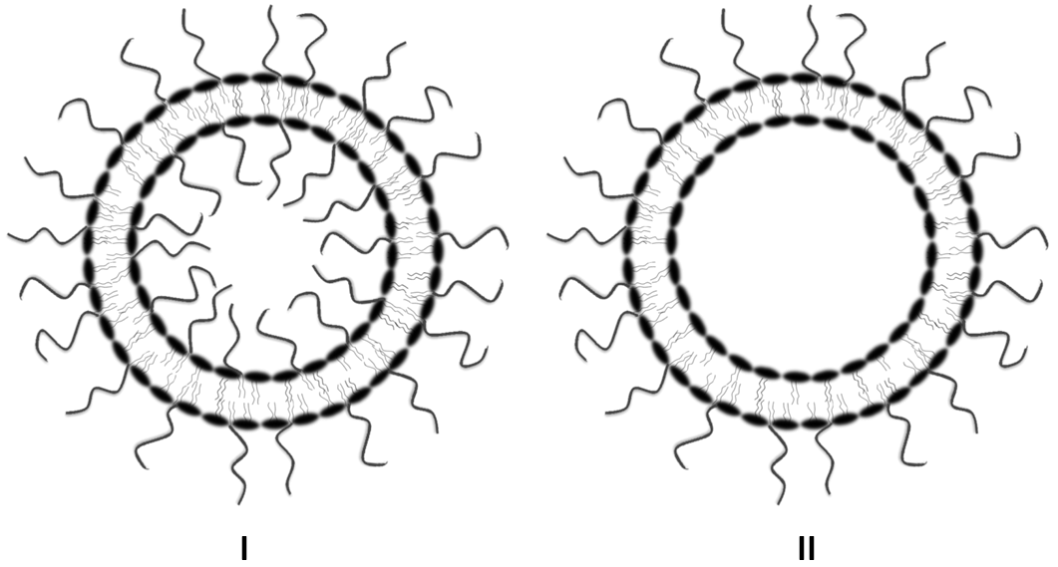
A comparison between liposomes pre-inserted and post-inserted with PEG-lipid. The post-inserted PEG-liposomes (**II**) offer several advantages over pre-inserted PEGylated liposomes (**I**), where the internal space is limited by the presence of PEG chains from PEG-conjugated lipid in the internal layer of the liposomes.

In the pre-insertion process, the lipopolymer is added to the lipid phase before the liposomes are formed by hydration with an aqueous phase. This is the most commonly used technique for the formulation of stealth liposomes [139,145]. The lipopolymer gets incorporated into the liposome structure, with a lipid portion inserted into the bilayer and the hydrophilic part populating the aqueous surfaces. Although this method seems relatively easy, there are some drawbacks attributed to the pre-insertion method. First, it requires an excess amount of lipopolymers, which, owing to the high viscosity, makes the extrusion process challenging [146]. Second, both the inner and outer sides of the lipid bilayer membrane are modified (Figure 3). The modification of the inner surface is undesirable, because not only does it occupy the valuable interior space in the vesicle, it also does not contribute to the steric stabilization of the liposomes [147,148]. Thus, the steric hindrance provided by the lipopolymers minimizes the real estate available for the encapsulation of drugs and biomolecules [147]. In addition, it has been found that internal lipopolymers (e.g., PEG-phospholipids) are susceptible to acid/base-catalyzed hydrolytic degradation in liposomes prepared for pH gradient-based active loading. The hydrolysate has the potential of compromising the active loading and retention of drugs inside such liposomes [146]. Equally important is the economic consideration of having the expensive, but essentially ineffective, internal presence of PEG-lipids, which does not contribute to the intended enhancement of the stability and circulation persistence of liposomes. The smaller the liposome size, the greater is the impact of internal PEG on each of the above outcomes. In the case of multi-lamellar liposomes, the magnitude of this wastage is even greater [147]. Furthermore, the pre-insertion technique is not ideal for the preparation of target-specific stealth liposomes with terminal antibodies, antibody fragments, peptides or other ligands.

### 6.2. Post-Insertion

The realization of the problems associated with the conventional PEG-phospholipid incorporation led to a technique of inserting PEG-distearoylphosphoethanolamine (PEG-DSPE) into the outer layer of pre-formed liposomes [149]. This useful technique is known as post-insertion or post-modification of liposomes and is beginning to attract applications in liposome-based drug and biologic delivery [146,147,150,151]. Reported first by Uster *et al.* [149] for the insertion of PEG-derivatized phospholipid into the pre-formed liposomes, in this method, lipopolymers are slowly added to the dilute suspension of the pre-formed liposomes at temperatures close to the *T*_m_ of the constituent lipids [146]. The insertion of the lipopolymer is a spontaneous process, which is driven mainly by the hydrophobic interaction of membrane lipids and the hydrophobic part of lipopolymers. In order to prevent the self-assembly of the amphiphilic lipopolymers, their concentration is maintained at a level lower than their critical micellar concentration (CMC). The thermodynamic barrier to insertion into the liposome bilayers is lower in the case of monomeric PEG-lipids than that in the case of self-assembled micellar PEG-lipids [150]. We recently provided confocal microscopic evidence of the stable presence of PEG on the surface of the liposomes post-inserted with HDAS-PEG [35]. We also demonstrated that PEG-lipid molecules desorb from the liposome surface upon dilution, resulting in gradual loss of the PEG-conferred stealth property [35]. Earlier, we reported the enhanced circulation persistence of liposomes PEGylated with post-inserted PEG_5000_-DSPE in a rabbit model. The PEGylation increased the presence of liposomes in blood at 24 h post-injection by approximately three-fold [147]. However, the likelihood of PEG-lipid insertion into the liposome surface is dependent on the PEG chain-length, where the incorporation is reduced as the PEG chain-length is increased [40].

As illustrated in Figure 3, the advantage of the post-insertion method is that the lipopolymers modify only the outer surface of liposomes, thereby keeping the internal space of the vesicle available for the accommodation of drugs or biomolecules. A number of comparative studies have demonstrated positive outcomes of the post-insertion over pre-insertion method. By monitoring the change in the zeta potential after PEGylation, Yoshino *et al.* showed that compared to the post-inserted liposomes, the pre-inserted liposomes took almost double the amount of PEG-lipid for a similar change in the zeta potential [146]. The additional consumption of the PEG-lipid in pre-insertion method is attributed to the presence of PEG-lipid in the internal lipid layer, which does not contribute to the change in the zeta potential. In addition, the anion exchange chromatogram of the pre-inserted liposomes exhibited relatively broad peaks compared to the post-inserted liposomes, suggesting that the pre-inserted liposomes have more heterogeneous surface properties than the post-inserted liposomes [146]. In another study, irinotecan-loaded, post-inserted liposomes showed higher circulation capability than that of their pre-inserted counterpart. Moreover, intravesicular PEG-lipid degradation was markedly inhibited in the post-insertion method [146].

### 6.3. Post-Modification by Chemical Reaction

The technique based on a chemical reaction between the polymer and liposome surface has been mostly used to modify liposomes for the purposes of targeted drug delivery, but not so much for enabling long-circulating liposomes. Nonetheless, a brief review of such technologies is relevant, because the same could be translated for PEG- or other polymer-modification of liposome surfaces. However, the inherent fragile nature of the liposome structure, solvent and reaction conditions, the difficulties in the separation of free reactants and modified product, the limited availability of aqueous reagents, aggregation and phase segregation and the possibilities of side reactions should be kept in consideration. The use of milder reaction conditions, such as those employed in “click” chemistry, are beginning to appear in the literature. For instance, liposomes incorporating polydiacetylene lipids terminated with alkynyl groups in their bilayer have been recently reported [152,153]. The exposed alkynyl groups are available to react with azide-functionalized polymers in a copper-catalyzed reaction. The resultant azide-alkyne cycloaddition could be applied for a rapid attachment of polymers with controlled composition onto the liposomes. After the reaction, the copper species and other small molecule reactants could be easily removed by treatment with EDTA (ethylenediaminetetraacetic acid) followed by size exclusion column chromatography. A variant of traditional azide-alkyne click chemistry is the UV-catalyzed reaction between thiol and alkyne groups [154]. Another potential method for chemically modifying the liposome surface is by oxime formation in a reaction between hydroxylamine and aldehyde [155]. Assuming the availability of hydroxylamine-terminated lipid and PEG-aldehyde, this chemoselective and bioorthogonal method could serve as an excellent alternative to post-functionalize existing liposomes in solution by simply mixing the two components without any other additives. Yet another mild reaction mechanism is based on the photoactivated conjugation of polymers to unsaturated lipid constituents of the liposomes. Used extensively for the preparation of polymerized liposomes [156], the application of this technique on liposome surface modification is dependent on the existence of polymerizable double bonds located at the end of the hydrocarbon tail of the surfactant or at their head groups.

## 7. *In Vivo* Consequences of the Surface-Engineering of Liposomes

Long-circulating or stealth liposomes have found clinical applications in therapeutic, as well as diagnostic medicine [157]. Once encapsulated, the drug or the diagnostic agent loses its inherent pharmacokinetic behavior and acquires the circulation kinetics, clearance and distribution characteristics of the liposomes [158,159,160]. However, the characteristics of the liposome itself in the biological milieu are determined by its surface and membrane properties, including bilayer fluidity, surface charge density, surface hydration and steric interactions with the medium [161]. As a particulate drug delivery vehicle meant for intravenous administration, the liposome surface mostly interacts with vascular endothelial cells and macrophages responsible for clearing the particulate materials. The goal of stealth technologies is to overcome the effects of the surface properties of the liposomes without pharmacologically affecting the clearance mechanisms of the body.

**Figure 4:**
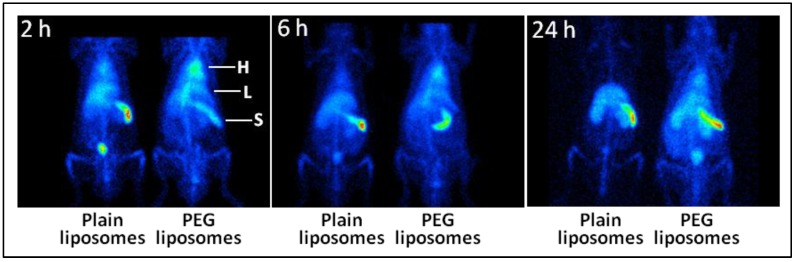
The effect of PEGylation on the circulation persistence of liposomes. As described elsewhere [35], liposomes consisting of DSPC (1,2-distearoyl-*sn*-glycero-3-phosphocholine)/Chol (cholesterol)/DMPG (1,2-dimyristoyl-*sn*-glycero-3-phosphoglyerol)/α-tocopherol, 45:44.8:10:0.2 mol%, were PEGylated with hexadecylcarbamoylmethyl hexadecanoate-PEG. The liposomes were labeled with Tc-99m, administered in rats, and the rats were imaged with a gamma camera over 24 h. As is evident from the heart (H) image signal, the PEG-liposomes remained in circulation even 24 h post-injection. The accumulation in the liver (L) and the spleen (S) was also lower in the case of PEG-liposomes, as compared to the plain liposomes.

After intravenous administration, the effect of the surface engineering of liposomes manifests itself in higher blood levels and lower MPS uptake. Using imaging technologies and liposomes labeled with radionuclide Tc-99m in a stable manner, we have provided the visual evidence of the effect of PEGylation on the circulation kinetics of the liposomes (Figure 4) [35]. The images clearly demonstrate that PEGylation significantly enhances the persistence of liposomes in circulation. At the same time, the uptake of PEGylated liposomes in organs responsible for particle clearance (liver and spleen) decreased [35]. Similar visual and quantitative evidence has been presented elsewhere [145,162,163,164]. However, the steric stabilization afforded by polymer coating is of finite duration, and even stealth liposomes are eventually cleared by MPS *in vivo*. In order to explain the finite nature of steric stabilization, we performed an *in vitro* desorption study, where the surface-bound PEG was assayed over time with respect to dilution [35]. We found that PEGylated liposomes are not completely invisible to complement protein and that PEG-lipid molecules gradually desorb from the liposome surface, resulting in a steady loss of the PEG-conferred stealth property [35]. The PEG desorption is a time- and dilution-dependent phenomenon and might also be occurring *in vivo*, where the rate of desorption could be higher because of the better maintenance of sink conditions and the presence of plasma [35]. The other unexplored explanation could be based on the opsonization mechanisms overwhelming the stealth strength of the PEGylated liposomes.

Prolonged systemic circulation provides the particles with an increased chance to extravasate into the target tissue, but it also enhances the drug exposure of other tissues, which may lead to toxicity [165]. For instance, patients administered with Doxil^®^ encountered severe skin toxicity [166]. In this regard, the utility of the PEGylated liposomes has been questioned from time-to-time. Studies have shown that PEGylation increases the concentration of liposomal doxorubicin in plasma, but offered no benefit in the treatment of tumors over plain liposomal doxorubicin [167,168]. It has been shown that only PEGylation of doxorubicin-loaded liposome does not improve doxorubicin accumulation and therapeutic efficacy in treating tumors, when compared with non-PEGylated doxorubicin-loaded liposomes [169].

It is also notable that although PEG modifies the pharmacokinetics of liposomes, it does not enhance their cellular uptake. However, the incorporation of a target-specific moiety, such as anti-CD19 on to the surface of doxorubicin-loaded PEGylated liposomes [170], can significantly improve their cellular uptake and resultant efficacy. Such advances in bioconjugation technology have enabled improvement in therapeutic and diagnostic targeting of stealth liposomes [171,172,173]. The availability of PEG-lipids with reactive terminal functionality, which easily reacts with target-specific biomolecules, such as peptides, proteins, ligands, antibodies and aptamers, has enabled such developments [2,171,172,174,175]. The most commonly used terminal functional groups in PEG-lipids include carboxyl, amine, hydrazide and maleimide. In one such example, DSPE-PEG-COOH was conjugated to an amine group of cyclic-RGD peptide using carbodiimide chemistry for the preparation of streptokinase liposomes targeting blood clots [176]. The targeted liposomes showed higher accumulation and increased thrombolytic activity at the site of the blood clots as compared to the PEG-liposomes without RGD ligand. A similar targeting ligand of RGD peptide was also reported for targeting the liposomes to α_v_β_3_ integrin in tumor neoangiogenesis [177]. It was found that the targeting of liposomes carrying DSPE-PEG-RGD was dependent on the extent of RGD and PEG loading. The ligand-integrin interaction increased with higher RGD loading, whereas it reduced with higher PEG loading [177]. Earlier, it had been demonstrated that higher PEG loading (10 mol%) of immunoliposomes causes steric hindrance and results in lower accumulation of targeted liposomes in the target tissue *in vivo* [178]. Thus, to achieve optimum specificity, it is important to fine-tune the ratio between the stealth imparting PEG and the targeting moiety at a molecular level. To address this issue, Li and Huang proposed the concept of “sheddable or cleavable PEG” for tumor targeting [179]. On the other hand, there are a few reports showing successful targeting of PEG-liposomes. For instance, Hong *et al.* reported a transferrin-targeted PEG-liposome preparation encapsulating PEG-hydroxycamptothecin for tumor targeting [180]. The investigators found that targeted liposomes circulated in blood for a prolonged period (*t*_1/2_ = 21 h, compared to 23 h for non-targeted liposomes), accumulated in tumor nine-fold more than the free drug and were about two-fold more efficacious in tumor suppression than the free drug [180]. For more in-depth understanding of stealth technologies with target-specific delivery of liposome-encapsulated drugs, the readers are referred to an excellent recent review [2].

## 8. Conclusions

While PEG-phospholipids are still considered to be the most attractive choice for surface modification of liposomes, new materials and technologies are beginning to show some impact. In this regard, recent reports on superhydrophilic polymers conjugated to a non-phospholipid anchor look quite promising. Nevertheless, the development of non-PEG/non-phospholipid lipopolymers is still in its infancy, and many regulatory and commercial hurdles will have to be overcome, even after successful demonstration of their significant advantages over PEG-phospholipids.
